# A Review on the Use of Imaging Biomarkers in Oncology Clinical Trials: Quality Assurance Strategies for Technical Validation

**DOI:** 10.3390/tomography9050149

**Published:** 2023-10-17

**Authors:** Stephane Chauvie, Lorenzo Nicola Mazzoni, Jim O’Doherty

**Affiliations:** 1 Medical Physics Division, Santa Croce e Carle Hospital, 12100 Cuneo, Italy; chauvie.s@ospedale.cuneo.it; 2 Medical Physics Unit, AUSL Toscana Centro, 51100 Pistoia, Italy; 3 Siemens Medical Solutions, Malvern, PA 19355, USA; james.odoherty@siemens-healthineers.com; 4 Department of Radiology & Radiological Sciences, Medical University of South Carolina, Charleston, SC 20455, USA; 5 Radiography & Diagnostic Imaging, University College Dublin, D04 C7X2 Dublin, Ireland

**Keywords:** imaging biomarker, clinical trials, magnetic resonance, nuclear medicine, Computed Tomography

## Abstract

Imaging biomarkers (IBs) have been proposed in medical literature that exploit images in a quantitative way, going beyond the visual assessment by an imaging physician. These IBs can be used in the diagnosis, prognosis, and response assessment of several pathologies and are very often used for patient management pathways. In this respect, IBs to be used in clinical practice and clinical trials have a requirement to be precise, accurate, and reproducible. Due to limitations in imaging technology, an error can be associated with their value when considering the entire imaging chain, from data acquisition to data reconstruction and subsequent analysis. From this point of view, the use of IBs in clinical trials requires a broadening of the concept of quality assurance and this can be a challenge for the responsible medical physics experts (MPEs). Within this manuscript, we describe the concept of an IB, examine some examples of IBs currently employed in clinical practice/clinical trials and analyze the procedure that should be carried out to achieve better accuracy and reproducibility in their use. We anticipate that this narrative review, written by the components of the EFOMP working group on “the role of the MPEs in clinical trials”-imaging sub-group, can represent a valid reference material for MPEs approaching the subject.

## 1. Introduction

A biomarker is a “defined characteristic that is measured as an indicator of normal biological processes, pathogenic processes or responses to an exposure or intervention, including therapeutic interventions” [1,2]. Biomarkers must be measurable, but can be numerical (quantitative) or categorical (either a quantitative value or qualitative data). To be applied in clinical practice they must be reproducible, linked to relevant clinical outcomes, and must demonstrate clinical utility. In healthcare settings (and in research), biomarker uses include screening for disease; diagnosing and staging cancer; surgical targeting and radiotherapy treatments; guiding patient stratification; and predicting and monitoring therapeutic efficacy and/or toxicity. Biomarkers can be also used as predictors of traditional endpoints (i.e., surrogate endpoints) such as treatment response and survival [2].

Imaging biomarkers (IB) are a special example of biomarkers where the indicator is derived from in vivo medical images providing an attractive choice for clinical use as they can be implemented and used as a real-time, non-invasive, cost-effective option. While extensive literature exists on the development of biological biomarkers, a roadmap for the definition of an IB is not adequately defined yet. Nonetheless, in particular settings, the question of how IB acquisition and analysis should be standardized, and how terminology should be harmonized have been addressed by numerous academic, clinical, industrial, and regulatory groups [3,4,5,6,7,8,9,10,11,12,13,14,15,16,17].

Despite some IBs being used extensively and others showing great clinical potential, there is only a limited number of them currently guiding the clinical decision-making processes in hospitals, while many IBs of potential interest are often confined to academic literature without a clear clinical route for implementation [18,19]. Many IBs have been evaluated on a limited cohort of patients, and in retrospective and monocentric studies. To become routinely used in the management of patients, an IB should prove to be a reliable measure used to test the clinical hypotheses within a multicenter research clinical trial.

A subtle characteristic of IBs is that the same biomarkers can be produced by imaging devices of different makes and models installed in different clinical sites. These devices are designed, approved, maintained, and operated to provide images that diagnostic radiologists, nuclear medicine physicians and other clinicians interpret, often with little need to accurately quantify the imaging data obtained. Usually, the quality assurance plans, carried out to optimize and assure adequate image quality, are performed for visual analysis in many cases (not all) with little focus on how the image quality will affect quantification, and little, if any, focus on the standardization of IBs though different sites.

The roadmap to carry the IB to clinical practice is required to follow different simultaneous validation routes, such as technical, clinical, and cost-effectiveness evaluations [18]. The technical evaluation requires that the IB is measurable precisely and accurately, and also that it is widely available in all geographical territories. In the clinical evaluation, the IB should demonstrate measurability of biologically relevant characteristics or be a predictor of clinical outcomes. Cost-effectiveness must also be considered because IBs must not only demonstrate an association with health benefits, but also demonstrate ‘value for money’ when compared with the use of other clinical information.

In the following sections we will discuss some examples of quantitative IBs (QIBs) used in clinical trials, the sources of error to be considered (limited to just the acquisition device) and the strategies carried out in multicenter clinical trials to reduce the IB variability. Although the IB can be a very broad concept considering the number of potential candidates, we restrict our analysis here to commonly used IBs in the imaging fields of Magnetic Resonance (MR), Computed Tomography (CT) and Nuclear Medicine (gamma camera planar imaging, SPECT, PET). It is important to underline how the use of IBs in clinical trials requires a broadening of the concept of QA; the entire chain has to be overseen, from data acquisition to data analysis, in order to adequately estimate uncertainties and, consequently, to draw conclusions consistent with the hypotheses through the estimated QIBs. This is certainly a challenge for the MPEs involved in clinical trials. We hope that this narrative review can provide valid support for those approaching the subject, as this is the aim of the manuscript.

## 2. Imaging Biomarkers

We initially aimed to perform bibliographic research by searching on PubMed for various techniques (e.g., “SPECT”) together with the words “quantitative”, “quality assurance”, “MPE”, “clinical trials”, “standardization”, and “imaging biomarkers”. This, however, produced a large range of IBs that were not generalizable enough for a broad review (i.e., a new IB specific to a single trial only), and thus we gave priority in the selection to those proposed by the consensus of many researchers, peer-reviewed publications from professional societies and associations, published clinical guidelines, and also on the basis of the authors’ experience. Many international initiatives have dealt with the definition, description, and evaluation of the usability of QIBs. These include the European Imaging Biomarkers Alliance (EIBALL), the Quantitative Imaging Network (QIN), and the National Cancer Imaging Translational Accelerator (NCITA) [19,20,21,22,23,24]. Biomarkers selected and described in this review are reported in many of the above initiatives.

A non-exhaustive list of imaging techniques that can be used to derive IBs is reported in Table 1, with the associated quality assurance (QA) testing, harmonization strategies, required dosimetry, and advanced analysis. The most frequently used IBs will be discussed in detail in the text. Where possible, suggestions for the optimization of the acquisition protocols and for data analysis will be indicated to achieve better accuracy and precision. These suggestions will hopefully be useful to MPEs and other professionals in the use of IBs in clinical trials.

### 2.1. PET/CT

PET/CT reporting has historically been based more on the extent of the disease than on the affinity of the tumor itself to the tracer used [25]. Within the next paragraph we limit the description to the IB of glucose metabolism provided by 18F-fluoro-deoxyglucose (18F-FDG) PET/CT, because of its prevalence in PET imaging, and on the understanding that other IBs provided by different radiotracers can be rapidly extrapolated from this one.

#### 2.1.1. Qualitative Scale

Discrete qualitative scale, where the areas of uptake are compared to normal, disease-free organs and tissues are used often in response assessment. The most commonly used references are the liver and the blood pool usually measured in a large vessel such as the aorta as in Deauville [26] and in PERCIST [27] metabolic response criteria. The application of a discrete scale requires training for the readers and a good inter-reader variability was demonstrated in the literature for Hodgkin lymphoma (HL) [28,29,30] and Non-Hodgkin lymphoma (NHL) [31,32]. Moreover, it is easy to perform in clinical settings, can be done with standard-of-care software, and only requires that reference uptake of different scans of the same patient remain within a tolerable limit.

On the other hand, it does not overcome the problem of visual analysis when two areas with the same uptake could be misclassified if they are surrounded by different backgrounds [33].

#### 2.1.2. Semi-Quantitative Metrics

Standardized Uptake Value (SUV) is the ratio of the decay-corrected concentration of activity in the tissue to the injected activity normalized to the patient’s body weight (SUV corrected for lean body mass (SUL) has been introduced recently [27] but has not been yet used extensively in clinical practice [3,34]) has been proposed to override this problem [31]. SUV moves PET image analysis from a discrete to a continuous scale, and SUV metrics are calculated inside a volume of interest (VOI) that could be:

- Maximum value within the VOI (SUVmax): simple to measure and provides information about the most active tumor foci. Drawbacks include a strong dependence on image noise as it corresponds to a single voxel measure.
- Average of all values in the VOI (SUVmean): less vulnerable to image noise, but depends heavily on the delineation method used for drawing the VOI [35].
- Maximum tumor activity within a 1 cm^3^ VOI in the most active part of the tumor volume [3,35] (SUVpeak): associated with the most active part of the tumor, but in a standard volume, being therefore less dependent from noise.
- Volume of the VOI itself, Metabolically Active Tumor Volume (MTV) measured in ml or the product of the MTV by SUVmean, Total Lesion Glycolysis (TLG): they evaluate the extent and aggressiveness of disease. MTV and TLG are marginally affected by noise but are dependent on the delineation method used for drawing the VOI [35].

MTV measurements are of crucial importance in pursuing a quantitative approach to PET; however, as of yet, the precision, accuracy, and repeatability of MTV and TGV have not been established, which are prerequisites of a good prognostic index.

#### 2.1.3. Kinetic Modelling

Although it is possibly the most accurate metric, kinetic modeling, which describes the delivery, retention, and utilization of any radiotracer under investigation, is difficult to perform since it requires long dynamic PET scans, personnel with specific knowledge, and dedicated software for the analysis. It is usually used in single sites to perform pharmacodynamics studies, in phase I-II studies, or in novel non-18F-FDG tracers and has, to date, limited application in routine clinical practice. Recent developments such as the implementation of whole-body PET scanners have enabled kinetic modeling without increasing patient scan time (a large limitation with traditional PET systems), and thus may translate kinetic modeling to clinical routine for a variety of oncology and non-oncology radiotracers [36,37].

#### 2.1.4. Sources of Errors, Optimization Strategies and QA

The sources of error in uptake evaluation can be attributed to specific factors such as the dependence on the PET/CT scanners, site procedures for patient preparation, data acquisition and post-processing. The former point may present issues with cross-calibration of PET/CT scanners whereas the latter are faced with standardization of procedures [38].

Cross-calibration of PET scanners and dose calibrators is mandatory to minimize uptake variability. Cross-calibration consists of the acquisition, as detailed by the PET/CT manufacturer’s manual, of a cylindrical phantom in which an activity of ^18^F, measured with the dose calibrator, has been inserted. A calibration factor is set on the PET/CT scanner in such a way that the activity measured by the scanner is the same measured by the dose calibrator. Since the dose calibrators are usually calibrated to 3–10% precision and there could be some errors in the filling procedure of the phantom, the accepted difference in clinical practice between the activities measured by the scanner and the dose calibrator is usually set to 10%, although may be set to a tighter control limit in clinical trials or may be radiotracer-dependent depending on half-life (i.e., assay of ^15^O-H_2_O).

Variability amongst different scanners, even in a controlled environment of a multicenter clinical trial can be large, and in some cases prove to be different up to 25% [39]. Efforts of cross-calibration through the measurements of different phantoms permit a variability of less than 10% in multicenter clinical trials [40,41] while 5% should be a good requirement to derive PET/CT-based QIBs [42,43].

A plethora of image reconstruction algorithms exist, and many differences arise when the measurements are carried out in small volumes, where loss of counts due to spill-out and partial volume effects occur [44]. The recovery coefficient curve is a figure of merit describing the ratio between real and measured activity varying the dimension of the volumes. Ideally, the ratio should be one with a 10% variation due to the scanner calibration as seen above. In reality, the ratio is one for larger volumes and decreases slowly when the dimensions of the volumes are smaller than 5–8 mL [45]. A NEMA/IEC phantom with hollow spheres, which can be filled with a known activity, can be used to tune the reconstruction algorithm. Several parameters of the reconstruction algorithm, such as the number of iterations, the number of subsets, and the width of the Gaussian smoothing filter can be adjusted to achieve the best recovery coefficients.

SUV measurement variation across PET/CT scanners in the range of 10–25% just as the consequence of the intrinsic variability of the instrument is commonly observed in the context of multicenter clinical trials [45]. Hence, the cross-calibration of PET/CT scanners and ancillary instrumentation is the first condition to achieve an accuracy in tracer uptake measurement of 5–10% [39]. Several programs for the cross-calibration of PET/CT scanners have been carried out in the recent years by imaging and oncology societies: the EANM (EANM) accreditation program for site of excellence carried out by EARL Ltd. (Vienna, Austria) [46], the UK NCRI PET Clinical Trial Network [40], the ACRIN program [43], the Clinical Trial Network of Society of Nuclear Medicine and Molecular Imaging (SNMMI) [47], the JSCT NHL10 trial in Japan [48] and the FIL Cuneo core lab in Italy [41].

Regarding the standardization of the acquisition parameters, various recommendations have been released by the US and European nuclear medicine associations [3,34,35] in order to provide minimal standards to guarantee an efficient comparison of PET/CT metrics acquired at different time points (intra-patient) and between different patients (inter-patient), either at a single site or across multiple sites [25,49].

### 2.2. Gamma Cameras

Several IBs are currently used in nuclear medicine such as left ventricular ejection fraction, renogram radiotracer clearance, thyroid uptake, and myocardial perfusion. They all rely on dynamic imaging to measure the change in the number of detected counts per frame. Similar to PET scanners, gamma cameras are susceptible to the same issues with variability in QIBs across scanners employing different detection technology, acquisition protocols, and image reconstruction parameters in multicenter clinical trials.

Standardization using phantoms for many of the clinical indications that can be scanned may be implemented, for example, a custom-built 3D printed myocardial perfusion phantom aimed at providing ground-truth validation of multimodal, absolute MPI applications in the clinical setting [50]. The phantom enables tracer kinetic measurement, including time–activity curve and potentially compartmental myocardial blood flow analysis. Phantoms for MPI quantification of ejection fraction and left ventricular volume have also been utilized to evaluate the dependence on collimator and reconstruction parameter choice, demonstrating the need for standardization when evaluating these QIBs in nuclear imaging [51].

A recent workgroup has aimed to summarize normal QIB values for MPI stress/rest using 99mTc and 201Tl perfusion agents as well as 123I-MIBG sympathetic imaging and included correction values for sites performing CT attenuation correction, demonstrating dependency on gender, ejection fraction, number of ECG gates, and volumes [52]. The work shows that appropriate quantification based on common normal databases and standard technology plays a pivotal role in clinical practice and more so for research where multicenter studies require standardization between scanning and resulting IB values.

In radionuclide angiography (RNA) using radiolabeled red blood cells, the calculation of left ventricular ejection fraction (LVEF) is the primary IB for cardiotoxicity assessment in chemotherapy regimens. Work has shown that depending on the acquisition of SPECT or planar imaging data, the LVEF value may not be equivalent due to technical reasons [53], and also when compared to cardiac MR using thresholds of 50 and 55%, there was misclassification of 35 and 20% of cancer patients, respectively [54].

Recently, in particular for bone scintigraphy, an analog of PET SUV has been proposed in SPECT [18], based on the inter-calibration between the activity calibrator and the SPECT system through the acquisition of a uniform phantom with known activity. A new imaging gamera-camera that will be commercially available in a few years, i.e., based on integrated cadmium-zinc-telluride detectors, will help in having more reliable quantitative indices also in SPECT, which would also greatly help in dosimetry application (see next paragraph).

Phantom standardization provides a means to assess the differences not only between scanners, but also between acquisition techniques such as SPECT and planar, and in cross-modality imaging such as PET and MR [55,56]. Similar phantoms exist for many other clinical indications for gamma camera imaging such as dynamic renograms [57], SPECT renal imaging [58], head and neck/thyroid imaging [59], and excellent review works have been published summarizing phantom availability and types, more commonly involving the use of 3D printing [60,61]. The 3D printing of radioactive phantoms serves to address certain limitations with nuclear medicine phantoms in general, allowing phantoms without inactive walls (thus contributing to lower partial volume issues) and more patient-specific and complex shapes [62]. Other initiatives include radioactive cryogel phantoms which allow the study of motion correction strategies in realistic lesion-simulating shapes that deform with movement [63].

#### 2.2.1. Radionuclide Therapy (RNT) Dosimetry

In the field of RNT, to achieve the desired prescribed dose and to estimate the absorbed radiation dose after administration of the radiopharmaceutical, accurate dosimetry is needed pre- and post-treatment. The quantification of radiation dose can be performed with the use of planar or SPECT imaging of post-therapy radioligands, in that patient images of variable radionuclide distribution should be ‘translated’ from three-dimensional count maps into maps of radioactivity. A careful quantitation procedure is required where a radioactive source (typically a point source, syringe, or large phantom) calibration with an accurately known activity is required to convert recorded counts to activity.

Currently, there is no consensus method for calibration; multicenter studies have aimed to harmonize quantitative imaging across a range of vendor systems with a specific focus to dosimetry applications for thyroid radioiodine therapies [64,65,66], 177Lu-based therapies [67] and 99mTc pretherapy for microsphere embolization therapies [68]. As image acquisition and reconstruction conditions can be radionuclide dependent in terms of the number of energy windows, appropriate scatter/attenuation correction, sensitivity to radionuclide, dead-time, and collimator blurring, societal guidelines have been produced for quantitative imaging of 177Lu [69], 131I [70,71], and 90Y [72] RNT radioligands. Advanced 3D voxel-based dosimetry cannot be achieved by planar imaging, yet planar imaging still serves as the main dosimetry method for RNT due to its ease of applicability. Uncertainty of the data due to the diverse range of variable physical and scanner parameters that can be adjusted remains complex, although recent EANM guidelines offer a framework to model the propagation of errors in the measurement process specific to the dosimetry chain of absorbed dose at the organ or tumor level, together with clinical examples of how this can be implemented [73].

#### 2.2.2. Sources of Errors and Optimization Strategies

At the time of writing, no guidelines for quantitative SPECT/CT systems harmonization have been published [74]. Many studies showed the need for harmonization of quantitative SPECT/CT scanners across centers [75,76], given the unavoidable differences in the calibration of these systems, in the reconstruction methods and in the image/data correction techniques being applied. Software from different vendors may also produce different quantitative results from the same SPECT system. Recent work has demonstrated that five clinically suitable parameters of image quality assessed by uniform phantoms (background calibration factor, total activity deviation, noise coefficient of variation, hot contrast, and recovery coefficient) may be useful for the assessment of system performance in terms of correct quantitative acquisitions of images (however, in a limited bi-center study) [77].

In RNT, many national and international efforts have been made towards scanner harmonization, with the overall aim of allowing repeatability and reproducibility in the calculation of absorbed radiation dose. Initiatives so far have even led to the setup of a quantitative imaging network [78]. A key reason for this is likely to be the legal implications of the European Directive 2013/59/Euratom, stating that radiation doses for therapy purposes should be “individually planned and their delivery appropriately verified” [79]. Thus, there remain many efforts for RNT that have not yet propagated to SPECT or planar imaging. Although the EANM have produced PET harmonization standards [80], a SPECT/CT harmonization pilot study is in progress but, at the time of writing, it has yet to be completed.

### 2.3. Magnetic Resonance

The definition and subsequent translation into the clinic of MR-based IBs is one of the important points of research in medical imaging of the last years. Considering the versatility of the MR systems, there are many strategies that allow us to estimate parameters that are promising IB candidates.

However, for the majority of them, standardization is still lacking because there are many confounding effects that influence the measurement and it is often difficult to establish accuracy a priori. It is important to remember what has been done so far by QIBA [15,81], which has promoted initiatives for the best usability of MR biomarkers. A recent American Association of Physicists in Medicine (AAPM) report illustrates the main biomarkers derived from MR data [82]. Below, we will summarize the main MR techniques that allow the estimation of useful IBs in oncology, describing the strengths and pitfalls of each one.

#### 2.3.1. Magnetic Resonance Spectroscopy (MRS)

MR spectroscopy (MRS) allows obtaining signals from different molecules (after suppressing water and lipids) due to the chemical shift produced by shielding the molecular electronic orbitals. Although MRS may be performed for different nuclei (Na, P, F, etc.), we limit the description here to hydrogen.

Routine MRS use has been hampered by the low concentrations of the molecules (metabolites) of interest, often of a few mM, which generate a signal-to-noise ratio (SNR) much lower than that of MR imaging. It is limited in the literature primarily to brain and prostate applications, where the primary metabolites of interest are N-Acetyl-Aspartate (NAA), Creatine (Cr), Choline (Cho), Lipids (Li), Lactate (Lac), followed by Glutamate, Glutamine, and Gaba in the brain, and Choline, Creatine, and Citrate in the prostate [83].

MRS acquisitions can be based on single-voxel or multi-voxel (Chemical Shift Imaging, CSI) sequences, both 2D and 3D. The most common pulse sequences are Point RESolved Spectroscopy (PRESS), which samples a spin echo, and STEAM, which samples stimulated echoes. The latter allows the use of very low TEs (<10 msec), which is useful for reducing unwanted T2 weighting effects, but has lower SNR. Recently a consensus paper has been published on the reporting of MRS methods and results, to allow an adequate assessment of MRS studies [84].

##### Sources of Errors, Optimization Strategies, and QA

The main source of uncertainty in MRS is the intrinsically low SNR ratio, approximately 10,000 times lower than typical MR imaging. Consequently, the acquisition needs careful control to enable high accuracy, efficient water and lipids suppression, and effects normally somewhat tolerated in routine MR such as the presence of iron or paramagnetic material, patient movement, require minimization. Other sources of uncertainty can be reduced by accurate shimming of the magnetic field (FWHM of the final water signal <15–20 Hz for MRS of the prostate, and <5 Hz for MRS brain at 1.5 T), and if manual second-order shimming is available it should be used to minimize local field in-homogeneities. Post processing can be improved by the use of a reproducible pipeline for signals fitting and performing a visual evaluation of the presence of artefacts. The quality of the measurement can also be checked, i.e., evaluating the amplitude of the residuals of the signal fits using, if possible, the so-called ‘Cramer-Rao’ lower bounds [85,86]. Uncertainty due to movement can be improved by acquiring MR imaging before and after MRS acquisition, to verify that the volume of interest is always located in the same position (i.e., that the patient has not made any gross movement).

In multicenter trials, it is essential to harmonize both the acquisition sequence (TE, TR, strategy to suppress the water signal, etc.) and the pre-processing and fitting procedure testing them with regular quality controls. To date, there is no QIBA recommendation in this regard; however, there are many examples of QA protocols for MRS [87,88,89,90,91].

#### 2.3.2. Dynamic Contrast Enhancement (DCE) Perfusion

DCE perfusion studies employ fast T1-weighted gradient echo (GRE) acquisition sequences repeated many times during the injection of paramagnetic contrast medium. The obtained dynamic images show the signal variation due to the contrast bolus in the arterial and venous vessels and, in the case of pathology, in the extravasation from the intravascular to the extravascular and extracellular space [92]. DCE perfusion is used in the clinic in a range of oncology applications: among others prostate [93], brain [94,95], and tissue sarcomas [96].

##### Sources of Errors, Optimization Strategies, and QA

Volume transfer coefficient (K^trans^), extravascular extracellular space volume fraction (V_e_) interstitium-to-plasma rate constant (k_ep_), and Integral Area Under the Curve (IAUC) are the most commonly estimated IBs obtained by fitting the kinetic model to the DCE signal [92]. The estimation of these parameters depends significantly on the acquisition strategy employed, which in turn, must be optimized considering the tissue and pathology under examination [97,98].

Within the context of clinical trials, uncertainty in DCE techniques can be reduced by the harmonization of the gadolinium injection procedure (concentration and bolus rate). The injection rate should be as fast as possible, the use of standardized GRE acquisition sequence in terms of field of view, TE, TR, resolution and tissue coverage, and harmonization of the timing resolution of a GRE sequence, even if using different accelerating techniques (parallel imaging, half Fourier, reduced K-space sampling, etc.). Usually, a time resolution of 1 image every 4–10 s is employed. Furthermore, the same total scan duration can be employed, as depending on the clinical purpose, different total scan times may be required (from 3–5 min up to >12 min to study processes with very low K^trans^) [97,98]. Temporal signal stability can be assessed considering the total scan duration, using homogeneous phantom measurements using standardized measurements (number of flip angles, etc.) in T1 sequences, as well as standardized B0 and/or B1 in-homogeneities corrections (especially in multicenter studies) [99] and standardized arterial input function (AIF) and pharmacokinetic modeling [100].

QIBA developed a specific phantom for the QA of DCE techniques [101], although many tests can also be performed with homogeneous phantoms with doped water (for example the assessment of the temporal stability of the signal).

It is noteworthy that there are many sources of uncertainty and variability in DCE perfusion and that much research is currently underway to standardize the technique and reduce the variability of the results. The work of dedicated groups of the Quantitative Initiative Network is currently underway [23].

#### 2.3.3. Dynamic Susceptibility Contrast (DSC) Perfusion

In DSC perfusion imaging (primarily used in brain imaging) very fast MRI acquisition sequences are acquired while a bolus of intravenous paramagnetic contrast agent is injected. The DSC contrast is due to the passage of the paramagnetic contrast agent, which generates a loss of phase coherence of the spins on the transverse plane and, consequently, a signal loss. Once the contrast medium has passed from the arterial perfusion bed to the venous one, if the blood–brain barrier is intact, the phase coherence of the spins is recovered and, consequently, the signal amplitude of the tissues under examination [92].

By analyzing the wash-in/wash-out curve of the contrast agent, it is possible to estimate the Cerebral Blood Volume (CBV) on a pixel-wise basis. In practice, only relative estimates of the CBV, rCBV, are used, as the calibration in absolute units is strongly affected by subject-dependent parameters that are difficult to estimate (for example the hematocrit and local AIF) [83,102]. rCBV is a widely used biomarker in brain MRI, whose value is related to tumor angiogenesis [103,104].

##### Sources of Errors, Optimization Strategies and QA

DSC acquisition protocols usually employ echo planar imaging (EPI) readings and spin and gradient echoes. The EPI-SE sequences have the advantage of being less affected by the distortions, but have a lower sensitivity to the contrast medium. For this reason, EPI-GE sequences are often preferred. Consequently, one of the main limitations of this technique is related to not being easily usable in regions of interest with high magnetic susceptibility (for example close to air/bone/brain interfaces) or in the presence of other iron/paramagnetic agents (e.g., hemosiderin accumulations or calcifications).

Similar to DCE imaging, uncertainties in clinical trials can be reduced by harmonizing the gadolinium injection procedure, GRE acquisition sequence acquisition parameters, and time resolution of GRE sequences. A typical time resolution of one image every 3–7 s is employed. Similarly, temporal signal stability should be assessed considering the total scan duration, using homogeneous phantoms, such as employing geometric distortion correction and contrast agent extravasation which can be evaluated on digital phantoms [100].

To date, there is no QA protocol to be performed on a consolidated phantom for DSC perfusion. It is, however, advisable to regularly evaluate the uniformity of the static magnetic field B0 and to check the stability of the signal during the acquisition of the entire time series (absence/presence of signal drift).

#### 2.3.4. Arterial Spin Labeling (ASL) Perfusion

Arterial Spin Labeling (ASL) uses the endogenous flux of water molecules to study perfusion. In ASL, two images are acquired: a control image and an image tagged via an additional selective radio frequency pulse positioned on a feeding artery for the volume of interest. This pulse, called the “tagging pulse”, labels the water molecules present in the arterial flow. Therefore, the difference between the tagged image and the standard one will contain only the signal of the water molecules present in the arterial vessels and corresponds to an arterial flow map [105,106].

Cerebral Blood Flow (CBF), which is a relative measure of arterial blood flow, is the most commonly used IB in ASL. ASL is used in the clinic in the diagnosis and follow up of various brain diseases, such as neurodegenerative diseases [107], multiple sclerosis [108], stroke [109], and in the diagnosis and follow-up of brain tumors [110].

##### Sources of Errors, Optimization Strategies, and QA

The absence of the need for contrast agents’ administration makes ASL a very promising technique, which however, has some limitations. Signal differences between the standard and the tagged image are low (≈1%) reflecting a low SNR. For this reason, high static field MR systems are necessary (3 T and above being optimal). There are many different readout and encoding strategies in ASL pulse sequences, the variety of which can provide relevant differences in multicenter studies where different systems with different software updates are involved. Therefore, similar tagging techniques and imaging pulse sequences (similar readout, k-space sampling, etc.) are required. It has been shown that the use of different readout techniques can lead to large differences in CBF [111]. Excellent examples of different ASL acquisition sequences can be found in the literature [112].

In multicenter studies, care should be paid to the positioning of the labeling plane/slab. At the moment, there is no QA strategy defined for ASL techniques and QIBA has not yet opened a profile on this topic [113]. However, there are visual methods of selecting image quality that have shown promising results [114].

#### 2.3.5. Diffusion-Weighted Imaging and Diffusion Tensor Imaging (DWI and DTI)

Many IBs can be estimated from Diffusion Weighted Imaging/Diffusion Tensor Imaging (DWI/DTI) acquisitions, the most common being the Apparent Diffusion Coefficient (ADC-logarithm of the signal obtained using at least two b-values, usually a small and a large one, normalized to the b-value itself). The b value measures the degree of diffusion weighting applied and depends on the amplitude and on the time of applied gradients. If the diffusivity of the tissue water molecules is spatially isotropic, it is not necessary to change the direction of the diffusion gradient, as the estimated ADC will always be the same. However, this is not the case in almost any organ in the human body and when using multiple directions of the diffusion gradient, it is possible to estimate another IB, the fractional anisotropy (FA). For the estimation of FA, at least six directions of the diffusion gradient are required, i.e., one DTI acquisition [115].

Other biomarkers can be defined using more complex models. For example, the Intra-Voxel Incoherent Motion (IVIM) model, which allows the estimation of slow, fast diffusion coefficients and perfusion fraction [116,117,118], the Kurtosis model, which allows the estimation of the corrected diffusion coefficient and of the kurtosis, or the stretched exponential model [119]. All these parameters are currently used as IBs in oncology on different organs: prostate, breast, liver, prostate, and uterus [120,121,122,123,124].

##### Sources of Errors, Optimization Strategies, and QA

The sources of variability and uncertainty that can influence the values of DWI-based biomarkers (ADC, etc.) are many and depend on several factors: the patient (breath, etc.), the sequence (TE, TR, number and choice of b-values, diffusion gradient directions, number of averages, spatial resolution, fat suppression effectiveness, correction for gradient field non-linearity), and the MR system (static field strength B0, gradient field non-linearity). The choice of the best acquisition strategy depends on the organ/tissue being analyzed and in particular, on the diffusivity of the healthy tissue and the type of tumor under analysis, on the degree of diffusion isotropy of the healthy tissue (for example white matter has high anisotropy and therefore it will be necessary to take this into account when optimizing an acquisition protocol), on the organ motion (for example the liver), and on the presence of sources of local static magnetic field in-homogeneities (for example, the air in the rectum in prostate imaging). Furthermore, the optimization of DWI sequences strongly depends on the chosen IB, that is, on the model chosen for the signal analysis. In particular, if the ADC is used, it is possible to limit the number of b-values (<5) with a medium value for the maximum b-value (=1000 mm^2^/s). To use IVIM diffusion models, it is necessary to sample the DWI signal even with low b-value acquisitions (<200 mm^2^/s) as the DWI signal is sensitive to rapid diffusive motions only in this range of b-values. On the contrary, if molecular hindrances are to be analyzed by means of diffusional kurtosis model, is necessary to increase the maximum b-value (>2000 mm^2^/s), without sampling low b-values DWI signal (<200 mm^2^/s). Details on the link between different water diffusivity regimes, DWI signal and b-values, can be found here [125].

Increasing the b-value implies a reduction in the SNR and an increase in the minimum achievable TE; therefore, the acquisition sequence must be modified accordingly. This optimization must also consider the type of MR system available: static field strength B0, type of receiver coil (number of channels, etc.), and the availability of imaging acceleration strategies (SENSE, Grappa, Compressed SENSE, Sparse sampling, etc.). Furthermore, if the tissue is strongly anisotropic, it is necessary to use an adequate number of directions of the diffusion-weighting gradient, in order not to introduce unwanted bias [126,127,128].

Discussing the optimization of DWI sequences for every organ is beyond the scope of this review, but we provide the main references for interested readers. In particular, the PIRADS guideline for the DWI of the prostate [122], the ISMRM guidelines for the DWI outside the brain [129], the EUSOBI guidelines for breast DWI and the QIBA profile [130]. Many studies underline the need to characterize the reliability of DWI measurements considering both the MR system and the acquisition sequence in multicenter studies, to avoid bias that may otherwise be difficult to detect [131,132,133,134,135,136,137].

It is, therefore important to define end-to-end QA protocols that allow evaluating the IB based on DWI on phantom, before using it on the patient. The easiest QA protocols to implement include the use of a water phantom or ice-water phantom, which allow for better temperature control, although it needs to be rebuilt after each set of measurements [133,136]. Another QA strategy may be based on the use of the NIST-ISMRM diffusion phantom (https://www.nist.gov/programs-projects/quantitative-mri (accessed on 2 August 2023)).

#### 2.3.6. Other MR-Based IBs

MR-based IBs less used in oncology or those with limited clinical use or experimental/unproven IBs are briefly introduced here. An important IB group is that related to relaxometry measurements, which allows a voxel-based estimate of T1, T2, and T2* tissue relaxation times. These IBs can be estimated with good spatial resolution and have found extensive use for the diagnosis of many diseases [138,139]. There is a QIBA profile dedicated to the use of relaxometry-based biomarkers, with indications also on the QA protocols to be adopted [82,140]. IBs based on CEST (Chemical Exchange Saturation Transfer—in which off-resonance RF pulses are used to saturate the signal of different molecules) are also proving popular. In particular, in oncology, the use of amide proton transfer (APT) contrast developed, which allows for the investigation of the tissue concentration of amide protons. APT is widespread especially for the study of cerebral gliomas, on MR systems with static field ≥3 T [141,142], although recent studies showed applications in extra-brain oncology [143].

Of note is the development of the first total-body MR system for Fast Field Cycling (FFC) MRI [144,145], a method which involves the transformation of the “static” field into a “variable” field. Another promising area of research for MR-derived QIBs is that of MR-LINAC hybrid systems, which have different technical characteristics with different challenges, and some references are indicated for interested readers [146,147].

### 2.4. Computed Tomography (CT)

Measurements of relative tissue density can be calculated for each pixel and numerically represented as Hounsfield units (HU) for comparison with reference tissues. HU is calculated based on a linear transformation of the baseline linear attenuation coefficient of the X-ray beam, where distilled water (at standard temperature and pressure) is arbitrarily defined to be zero HU and air defined as −1000 HU.

Specific organs can be highlighted by variations in the resulting gray tone scale (windowing and leveling). As in MRI, CT scans are often performed after the administration of timed doses of intravascular contrast media to enhance the differences between adjacent structures. The use of the HU to measure tissue density has aided radiologists in the diagnosis of a wide range of diseases such as any incorporating bone mineral density changes, fatty liver diagnosis, evaluation of pulmonary nodules, bone quality before/after mechanical intervention, cyst/tumor differentiation, coronary artery calcification, and kidney/gall stones amongst a host of other uses.

HU accuracy is extremely important from the perspective of quantification, and even when used directly in differential diagnosis criteria. For example, in the management of adrenal masses, clinical guidelines detail that an adrenal mass with CT density >10 HU has 100% sensitivity for the detection of adrenal malignancy (confidence interval 91–100%), and that HU = 0 is likely to suggest a benign adenoma [148]. Such criteria involving HU thresholds are commonplace in the management of other diagnostic criteria such as breast cancer [149], intracranial hemorrhage [150,151] and Agatston calcium scoring [152] to name but a few, and demonstrate an innate dependence on the accuracy of HU as an imaging biomarker.

#### 2.4.1. Sources of Errors, Optimization Strategies, and QA

CT quality control of the HU scale has been prescribed according to a multitude of worldwide professional bodies such as the ACR (United States) [153], IPEM (UK) [154], and ACPSEM (Australia) [155]. These can be implemented as a minimum standard for both clinical operations and any trials requiring accurate CT quantification. Modern routine QC have fully automated evaluation routines, and initiatives involving automation of more complex ACR testing (i.e., other than image noise, CT number of water and image uniformity, for example) have shown to lead to fast action in a local institutional setting and may be useful from the perspective of QC monitoring in a clinical trial [156].

HU number accuracy and acceptable tolerances (i.e., ±4 HU as defined by ACR or ±10% as defined by IPEM) are well defined for specific exposure criteria and materials (i.e., water, air, acrylic, Teflon, etc.) to ensure scanner repeatability under fixed conditions. As polychromatic energies comprise the conventional CT X-ray beam, X-rays of differing energies will result in different tissue absorption properties and hence, different HU values. In modern conventional CT, this raises an issue that HU values and iodine quantification are dependent on the user-defined scan parameters [157]. Furthermore, iodinated contrast media show higher attenuation levels at lower X-ray tube voltage owing to higher photoelectric effect and lesser Compton scattering. With the continued drive towards lower radiation dose from each examination, and the improvement of scanner design (i.e., tube current/tube potential modulation, dynamic bow-tie filter design, detector quantum efficiency, software denoising algorithms), many imaging strategies optimize their imaging protocols to reduce the mA which can in turn affect the HU value bias (i.e., the value between a lesion and nearby background tissue). Recent work developed a theoretical framework to derive the quantitative relationship between HU bias, exposure level, and HU contrast on a clinical and experimental CT system, demonstrating in a phantom model (with different material inserts) that positive and negative bias can be observed depending on the contrast difference between a targeted ROI and its surrounding background tissues over a range of mAs from 60 to 500 [158].

The choice of image-reconstruction algorithm (especially with iterative reconstruction becoming more prevalent in CT) also plays a part in the accuracy of HU values, with standardization essential for clinical comparison in multicenter studies. Previous work evaluated this in 36 scanners detailing manufacturer dependencies that may need to be considered in the scope of a multicenter trial [159]. Iterative reconstruction over traditional FBP reconstruction also plays a part in the HU accuracy, primarily in how the algorithms handle noise reduction, which vary from vendor to vendor [160].

Accurate HU values are also subject to modification by the effects of beam hardening [161]. Although beam-hardening correction schemes are implemented in clinical scanners, in most cases the correction is calibrated only with a specific body size and kVp as HU values still change if the body size is varied [162]. A system calibration or correction scheme for all body sizes is required in order to remove the size-dependent variations of CT numbers, and not yet been implemented.

#### 2.4.2. Other CT-Based IBs

Given the dependence of the HU on energy, the use of HU as a quantitative diagnostic parameter is limited in Dual Energy CT (DECT). With the recent release of advanced clinical CT systems such as photon counting CT (PCCT), more accurate multi-energy imaging has become a possibility. This technology presents many clinical advantages in terms of higher spatial resolution, and lack of electronic noise, equal weighting of lower and higher energy photons, but also with the potential to remove the dependency of HU values on tube kV due to inherent spectral imaging in every scan [163,164]. Advanced reconstruction algorithms provide IBs such as those that remove calcium by combining material decomposed images with mono-energetic and virtual non-contrast images [165], and those that conversely remove iodine and leave calcium contributions [166].

#### 2.4.3. Radiotherapy CT

Given the precedence of CT in Radiation Oncology, the largest clinical work area in Medical Physics, it serves to dedicate a specific part of imaging to this growing field. In photon radiation therapy, HU from CT scans is further converted into values for mass density (MD) or relative electron density in water (ρe’), in order to calculate planned dose distribution from a treatment schedule. This is a crucial measure in radiotherapy treatment planning for inhomogeneity correction, especially in high-energy photon and electron beam therapy in anatomic areas where electronic equilibrium is not well identified.

Most radiotherapy institutions register the CT number calibration table in the treatment planning system, by scanning commercially available tissue-equivalent material and obtaining a HU-ρ_e_ curve. The CT number calibration table is easily obtained using these tissue-equivalent phantoms; however, the manufacturer determines tissue-equivalent materials. Consecutive CT calibration tables must be created from the discrete CT number for each tissue. The most common phantoms to use are the Gammex and the Catphan series of phantoms—both of which contain inserts of gold standard attenuation characteristics, although many sites have created their own [167].

##### Sources of Errors and Optimization Strategies

The HU to ρe curve is dependent on the phantom model [168], where the differences for the larger HU values can be attributed to the effective atomic number of the materials. Much work has investigated a stoichiometric calibration method to overcome the problem of using tissue substitute materials (i.e., not of the same density as human tissues) by predicting the HUs of real body tissues. This research has attempted to compare CT number calibration with a tissue-substitute CT number calibration and compare the parameterization models of the stoichiometric process [169,170,171,172]. Some studies have reported that high-atomic-number material, such as barium (Z = 56), is not appropriate for CT number calibration [170,173]. A conventional CT number calibration is performed using nominal CT numbers for air (−1000 HU) and water (0 HU). However, there are CT scanners in which the CT number for air is not −1000 HU, so, as fitting parameters in a conventional stoichiometric CT number calibration are forcibly determined, using the CT number −1000 HU for air, a CT number calibration error may be introduced—particularly for low-density tissues such as lung [174]. Furthermore, a required minimum number of materials for stoichiometric CT number calibration has not been established.

It is appropriate to compare the CT calibration table with standard tissue data from the CT number calibration audits of a third party, simply because some tissue-equivalent materials differ from standard tissues. A standard tissue’s CT numbers are calculated using a stoichiometric CT number calibration based on the elemental weights of the standard tissues, obtained from the International Commission on Radiological Protection Publications and the International Commission on Radiation Units and Measurements Reports [175,176].

## 3. Discussion and Conclusions

A recent guidance document on imaging clinical trial endpoints from the Food and Drug Administration (FDA) in the United States mentions [177]: “Although the medical practice of diagnostic imaging already follows many standardized procedures, we recommend that some trials augment these existing standards to create trial-specific imaging process standards.” In the context of an imaging study performed by a Radiology/Nuclear Medicine department, many of the technical process standards (i.e., software availability, specific equipment quality control, image curation/archiving, equipment performance features, etc.) will fall to a medical physicist as the person most likely to have enough scientific and technical know-how to play a key role in developing an imaging charter or clinical trial protocol [178]. Most multidisciplinary trials (including industry-sponsored trials) likely require guidance/consultancy from MPEs on the best imaging parameters and techniques and how standardization and harmonization can be achieved with other sites (if implemented) in the imaging network. Their role In trials and clinical imaging and patient care in general can be summarized in 12 expectations of experience [179]: scientific perspective, quality/safety assurance, regulatory compliance, technological awareness, performance monitoring, equipment commissioning and optimization, translational practice, vendor cooperation, and research.

This review dedicated to QIBs is written following the point of view of the MPE. The sources of uncertainty that limit QIBs accuracy and reproducibility are reported, indicating, where possible, the strategies to optimize the estimations. From this point of view, this review has two limitations. The first is that not all the systems/techniques that can be used to estimate QIBs are described, although they involve the MPEs as well, due to complexity and too broad a scope. For this point, we reinforce in particular hybrid systems such as MR-LINAC and PET-MR, radiomics, artificial intelligence and multi-modal imaging. These topics represent important fields of research and development in oncology clinical trials, as evidenced by numerous recently published articles, including some position papers from international scientific societies [180,181,182,183,184,185,186,187,188,189,190,191,192,193,194,195,196,197,198,199]. The second is that it was not possible to follow a rigorous selection criterion to include the articles, given the high number of manuscripts published in recent years on QIBs. However, this limitation is partially exceeded because we have taken care to consider the most important sources, such as, for example, the QIBA of the RSNA and the AAPM [81,82,87,101]. Regarding the international societies active in the field, it is also important to mention the publications, some very recent, of the Open Science Initiative for Perfusion Imaging (OSIPI) group of the International Society for Magnetic Resonance in Medicine (ISMRM), which focuses on perfusion MRI [200,201,202].

A specific mention should be made to the emerging field of Radiomics in medical imaging, which aims to enhance clinical decision making through the quantification of textural information and mathematical analysis of the spatial distribution of signal intensities and pixel interrelationships. Given the field of radiomics is currently undergoing rapid expansion in all modalities in medical imaging, professional societies have aimed to provide guidance and best practices on how to effectively implement radiomics results into clinical data analysis, for example, standardizing image processing, harmonizing image-acquisition techniques, how to incorporate biological data, and image data normalization and segmentation. Examples include the European Society of Radiology (ESR) endorsed recommendations on using radiomic data in clinical trials [24]. This framework represents a cutting-edge consensus pathway determined by imaging experts from the European Society of Radiology EIBALL (European Imaging Biomarker ALLiance), the EORTC (European Organisation for Research and Treatment of Cancer) Imaging Group, and QIBA. Recently, a joint EANM/SNMMI best practice statement for data specific to Nuclear Medicine has also been published [203].

Multicenter studies in radiomic analysis are required in order to improve statistical accuracy, especially important in rare diseases [204]. A recent intercomparison by the Image Biomarker Standardization Initiative (IBSI) between 25 institutions of radiomics processing tools using CT, PET and MR data aimed to provide benchmarking for radiomics analysis software [205]. Although initially, the intercomparison demonstrated a weak consensus, by the end of the study the sites were able to produce and validate a set of consensus-based reference values for radiomics features. This work standardized 169 of 174 features and provides a benchmark for which compliance with the IBSI standard can be checked for any radiomics software.

Medical imaging, and biomarkers too, has to deal with the new technology exponentially growing in all fields of science and everyday life—artificial intelligence. Being outside the scope of this paper to discuss the hundreds of applications (several good reviews are available, for example [206,207,208,209,210,211]), what we want is to point out that in general AI could help to develop stable and reliable bio-markers. AI, and in particular deep learning is used in the image reconstruction algorithm of almost all imaging modalities (CT, MRI, PET). AI has been applied in assisted readings (e.g., in second readings for mammography and lung cancer screening). AI is also used to combine imaging readings to clinical information and patient characteristics to develop diagnostics and prognostics tools. We indeed expect that several all-comprehensive AI tools will come out to help clinicians in everyday life. But before going into clinical practice, in a specific hospital environment, which may be different from the one tested for marketing approval (e.g., different acquisition protocol, different patient settings), acceptance testing of AI should be carried out. And indeed, we think that MPE, with their long-standing experience in quality assurance, could definitely help with this.

The current strategies carried out to provide a reliable IB are based on two pillars: the accreditation of clinical imaging laboratories as being competent for measuring it, and the standardization and harmonization of its measurement during the clinical trial. The latter in particular is an important step towards improving technical performance in multicenter studies, but must reflect widely sought academic consensus, become adopted by international societies, and receive the backing of funders, industry and regulators for such accreditation to have value. Such an approach has been for example carried out since 2007 by QIBA, an initiative aimed at improving the value and practicality of quantitative imaging biomarkers by reducing variability across devices, sites, patients, and time.

## Figures and Tables

**Table 1:** Imaging techniques that can be used to derive Quantitative Imaging Biomarkers (QIBs). The table provides suggestions for the implementation and the use of Quantitative Imaging Biomarkers in clinical trials. The table is intended as a non-exhaustive guide for the reader, in which an attempt has been made to highlight the most relevant points for practical purposes. QIBs are reported following the order of the systems needed to estimate them (starting with PET, then SPECT, etc.). A detailed description with references of the QIBs shown in the table is given in the main text.

Technique	Pre-Trial Quality	Hints on Harmonisation	On-Trial QA	Dosimetry	Patient (Staff) Safety	Advanced Analysis
Positron Emission Tomography (PET) qualitative analysis	Image quality phantom (NEMA/IEC/ACR phantom); analyisis of recovery coefficient curve SUV accuracy validation	Requirements on: administered activity, uptake time, fasting state, blood glucose level	Patient image quality: visual assessment OK, SUV readable	Activity check	absolute exclusion criteria for pregnancy, relative exclusion criteria for breastfeeding	
PET quantitative analysis	Image quality phantom (NEMA/IEC/ACR phantom); analyisis of recovery coefficient curve, requirement for recovery coefficient at one data point SUV accuracy validation	Requirements on: administered activity, uptake time, fasting state, blood glucose level,	SUV validation on the day of patient acquisition Patient image quality: normal organs (e.g., liver or blood pool) SUV range within limits		absolute exclusion criteria for pregnancy, relative exclusion criteria for breastfeeding	Use of semi-quantitative metrics: SUVmax/peak, DeltaSUV, MTV, TLG, radiomics, and full kinetic analysis.
Q-SPECT	Quantitative SPECT calibration Accuracy of administered activity	Jaszczak/other phantom comparison (quantification) Requirements on: administered activity, uptake time, patient preparation (fasting, thyroxine, beta blockers, etc.)	Patient image quality: visual assessment OK, HU readable	on trial check that injected activity is within ±5% of trial-specified activity. Audited post trial by sponsor/institute	absolute exclusion criteria for pregnancy, relative exclusion criteria for breastfeeding	Quantitative metrics: Activity concentration (MBq/mL), absorbed dose (Gy),
Computed Tomography (CT)	ACR/Gammex phantom for HU accuracy	Requirements on dual energy protocols and spectral imaging Noise matching between scanners	Patient image quality: visual assessment OK, HU readable	CTDI/DLP accuracy (SSDE as per IEC standard 2019)-calculated different ways	Exclusion criteria for pregnancy and contraindications for iodine contrast	Use of quantitative metrics: iodine/calcium concentration, material decomposition. Semi-quantitative metrics: ECV, ctFFR, radiomics kinetic analysis (perfusion studies)
MR Spectroscopy	Depends on the study goal and sequence characteristics (single voxel, CSI, relative or absolute quantification, J-editing). Pre-trial phantom acquisition should be performed to assess intercenter accuracy.	Acquisition standardization across multiple MR systems. The optimization strategy depends on the metabolite/s of interest and on the organ (usually brain and prostate).	Online assessment of spectral quality using quick preprocessing and fitting. In [REF Gulin O] the reference values for brain hydrogen MRS are indicated.		MR-related safety aspects, exposure to static and variable magnetic and RF fields	Verification of the consistency of the final data. Final estimate of signal amplitude uncertainty. In multicentre studies, the reproducibility of the data should be assessed, also considering the different pre-processing strategies. Criteria must be established to discard unreliable spectra, (Cramer–Rao lower bounds)
Dynamic Contrast Enhanced (DCE)-T1 weighted Perfusion MRI	QIBA developed a QA protocol for quantitative DCE-MRI	Use a similar gadolinium injection procedure. Acquisition standardization is mandatory to reduce the variability of estimation. Assess temporal signal stability. Homogeneous time resolution should be employed. If T1 maps are used to account for T1 relaxation time of tissues, acquisition strategies dedicated to T1 estimation should also be standardized. If Bo and/or B1 in-homogeneities corrections are used, their effect should be assessed on phantoms	Visual assessment of image quality.		MR-related safety aspects, exposure to static and variable magnetic and RF fields and gadolinium contrast agent administration	Many different models to estimate quantitative biomarkers. Some include corrections for B0 and B1 in-homogeneity, and for the T1 relaxation time of the tissues. The most common DCE-based biomarkers are K^trans^ and IAUC (QUIBA established that both can be measured on a 1.5T system with an inter-subject variability < 20%)
Dynamic Susceptibility Contrast (DSC)-T2* weighted Perfusion MRI	QIBA developed a QA protocol for quantitative DCE-MR.I B0 inhomogeneity should be checked, considering that echo planar imaging is usually employed	Use a similar gadolinium injection procedure. Acquisition standardization is mandatory to reduce variability of estimation. Assess temporal signal stability. Minimize distortion artefacts due to EPI. Homogeneous time resolution should be employed. Where possible, use 3D gradient distortion correction map.	Visual assessment of image quality. B0 field in-homogeneity routine check		MR-related safety aspects, exposure to static and variable magnetic and RF fields and gadolinium contrast agent administration	Many different models to estimate quantitative biomarkers. The most used is relative Cerebral Blood Volume (rCBV)
Arterial Spin Labelling ASL Perfusion MRI		Acquisition standardization is mandatory to reduce the variability of estimation. Consistent and reproducible positioning of tagging slab.	Visual assessment of image quality.		MR-related safety aspects, exposure to static and variable magnetic and RF fields	Difference between “tagged proton” flow sensitive image and the same image without proton tagging. The most used biomarker is Cerebral Blood Flow (CBF)
Diffusion Weighted Imagin—-DWI	Several protocols and phantoms have been developed for pre-trial DWI QA DWI-based biomarker, such as ADC, accuracy, and spatial in-homogeneity, has to be assessed before starting the study.	Sequence standardization is mandatory to reduce variability of estimation. Before starting the study, it is necessary to estimate the parameters of interest (for example ADC) using a dedicated temperature-controlled phantom. TR should be long enough to reduce T1 weighting, TE short enough to minimize T2 weighting. Parallel imaging should be employed to minimize eddy currents. The number and the values of b-values and the number of diffusion gradient encoding directions depend on the organ and the pathology. Where possible, gradient non-linearities correction should be employed	Constancy assessment: pre-trial phantom measurements should be repeated		MR-related safety aspects, exposure to static and variable magnetic and RF fields	The most used DWI-based biomarker is ADC. Other biomarkers, D, D* and perfusion fraction, can be estimated using IVIM model. The use of these models requires acquisition sequences with different b-values and different encoding directions of the diffusion gradient.
Relaxometry	Relaxation times (T1, T2, T2*) accuracy and inter-center reproducibility should be assessed by phantom measurements. Several phantoms have been developed for this purpose (ISMRM-NIST www.nist.gov/programs-projects/quantitative-mri (accessed on 15 July 2023), Eurospin T05 phantom, Diagnostic Sonar LTD, Livingston, Scotland) Spatial uniformity of the relaxation times should also be assessed	Acquisition standardization is mandatory to reduce variability of estimation. If B0 and/or B1 in-homogeneities corrections are used, their effect should be assessed on phantoms especially in multi-centric studies	Constancy assessment: pre-trial phantom measurements should be repeated		MR-related safety aspects, exposure to static and variable magnetic and RF fields	Different strategies can be employed to evaluate T1, T2 and T2*

## Data Availability

No new data were created or analyzed in this study. Data sharing is not applicable to this article.
